# Split Daily Oral Iron Dosing Enhances Correction of Iron-Deficiency Anemia in Rats

**DOI:** 10.1155/anem/9976840

**Published:** 2025-06-28

**Authors:** Mohamed M. El-Kady, Nermeen Bastawy, Mohamed Amin, Soha Elmorsy, Olfat Shaker, Abeer Mostafa, Eman Hassan Nadwa, Marwa Abdel-Rahman

**Affiliations:** ^1^Department of Medical Pharmacology, Faculty of Medicine, Cairo University, Cairo, Egypt; ^2^Department of Medical Physiology, Faculty of Medicine, Cairo University, Cairo, Egypt; ^3^Department of Medical Biochemistry and Molecular Biology, Faculty of Medicine, Cairo University, Cairo, Egypt; ^4^Department of Pharmacology and Therapeutics, College of Medicine, Jouf University, Sakaka, Saudi Arabia

**Keywords:** anemia, ferritin, hemoglobin, hepcidin, iron, split-dose of iron

## Abstract

Iron deficiency is the leading cause of anemia worldwide. Single oral iron daily supplementation is usually unsatisfactory. We hypothesize dividing the oral iron dose may improve the anemic parameters. To test this hypothesis, forty male Wistar rats were evenly assigned to the following groups (*n* = 8): Control nonanemic or anemic groups. Anemia was induced by repeated phlebotomy from the orbital plexus under anesthesia for 4 weeks. The anemic rats either received no treatment (IDA group) or received a total oral iron supplementation (7.1 mg/kg/day) for 1 week. Iron was administered in different treatment regimens: single dose per day (IDA-Fe-sid group), twice per day (IDA-Fe-bid group), or thrice per day (IDA-Fe-tid group). The hemoglobin concentration, hematocrit values, total iron-binding capacity (TIBC), and serum levels of iron, ferritin, and hepcidin were measured to assess the anemia. The results showed that administration of iron in divided doses for 1 week exerted significant restorative effects on the measured anemia parameters, unlike the single daily regimen. In conclusion: oral iron supplementation in divided doses increased the oral iron bioavailability; therefore, it may be more efficient in improving the anemia parameters than a single dose in short-term treatment of IDA. In clinical practice, dividing the total large iron dose into multiple smaller doses may correct IDA more quickly, especially in patients who cannot tolerate a single large dose.

**Summary**

• Iron deficiency anemia continues to be a worldwide health problem.
• Adequate iron intake is hindered by poor oral iron bioavailability.
• Dividing the iron dose significantly improved anemic parameters in moderate iron deficiency anemia in rats.

## 1. Introduction

Anemia is defined as a hemoglobin concentration of less than 12 g/dL in women and 13 g/dL in men. It affects approximately one-third of the world's population, with half the cases being due to iron deficiency [1]. The typical laboratory findings of iron deficiency anemia (IDA) are a depletion of serum ferritin and iron, high total iron-binding capacity (TIBC), and generally a low mean corpuscular volume [2]. Hepcidin, the liver peptide hormone, a major factor in iron homeostasis, also gets substantially suppressed in IDA [3].

A wide variety of adverse health outcomes are associated with IDA, including immune dysfunction, Helicobacter pylori infection, reduced clinical outcome in patients with cardiac diseases, cognitive decline, and impaired thermoregulation [4]. Severity of anemia is graded according to the hemoglobin levels, ranging from mild anemia (hemoglobin concentration between 10 and 10.9 g/dL) to severe anemia (hemoglobin level below 7 g/dL) [5].

The etiology of IDA is usually linked to an imbalance between iron loss and iron absorption [6]. Chronic blood loss is a crucial cause of IDA, as each milliliter of blood contains about 0.5 mg of iron. Although compensatory mechanisms can enhance iron absorption, they are unable to keep up once iron loss exceeds 5 mg per day [7].

Oral iron supplementation is the first-line treatment in the management of IDA. Oral ferrous sulfate preparations, containing 60 mg elemental iron, are usually prescribed one to three times a day [2]. Parenteral iron is sometimes required in cases of patient intolerance or therapeutic failure [6]. However, use of parenteral iron is limited because of cost and hypersensitivity reactions [8].

The iron transporters are primarily located in the duodenum and jejunum [9]. The small available surface area for absorption and the short transit time (30 min) confine free iron absorption [10].

Physiologically, oral iron bioavailability is very low (5%–25%) [11]. The divalent metal transporter 1 (DMT1), which is responsible for iron absorption at the luminal border of the enterocytes, shows a saturable kinetics (also called zero-order) [12, 13]. One way of overcoming this problem is to increase the time of exposure available for the drug absorption as long as no value will be attained from increasing the dose. The current study examined the effect of increasing oral iron dosing frequency while retaining the same total daily dose in order to maximize duodenal iron exposure time, thus increasing the fraction absorbed.

## 2. Materials and Methods

### 2.1. Drugs

Ferrous sulfate syrup (Haemojet, 10 mg elemental iron/mL. pharma company, Egypt).

### 2.2. Animals

This study was an experimental study conducted over 5 weeks. Forty adult male Wistar rats, aged 8 weeks (200–230 g), were housed under a 12-h day/night cycle (lights turn on at 7:00 in the morning) in an air-conditioned room (23 ± 2°C). Animals had free access to commercial laboratory feed and water ad libitum. The experiment protocol complied with the ARRIVE guidelines and approved by the Institutional Animal Care and Use Committee (CU-IACUC), Cairo University (CU-III-F-44-21).

### 2.3. Induction of IDA

The rats were subjected to biweekly bleeding (3 mL) from the orbital plexus (alternating eyes) under isoflurane anesthesia for 4 weeks [14]. This procedure was expected to decrease the mean baseline hemoglobin by about 30% based on a previous pilot study conducted by the authors.

### 2.4. Animal Groups

Rats were adapted to laboratory conditions during the first 3 days and then were randomly allocated to one of five groups (8 rats/group) as follows (Table 1): A control group of normal nonanemic rats (Control), an iron-deficient anemic nontreated group (IDA), and three anemic groups were treated with oral iron preparation as follows: once daily (IDA-Fe-sid), twice daily (IDA-Fe-bid), and three times daily (IDA-Fe-tid). Iron syrup (Haemojet) was administered by gavage for 1 week immediately after anemia induction. Control and iron-deficient anemic nontreated (IDA) groups received distilled water. The iron-treated single daily dose group (IDA-Fe-sid) received 7.1 mg/kg elemental iron [15], equivalent to a Haemojet dose of 0.7 mL/kg. The iron-treated twice daily dose group (IDA-Fe-bid) received a Haemojet dose of 0.35 mL/kg at 7:00 a.m. and 7:00 p.m. The iron-treated thrice daily dose group (IDA-Fe-tid) received a Haemojet dose of 0.23 mL/kg three times per day at 7:00 a.m., 1:00 p.m., and 7:00 p.m. Haemojet doses were diluted with distilled water to make an equal volume of 0.3 mL/animal.

The total daily iron dose administered to all treated anemic rats was 7.1 mg/kg, selected based on standard dose conversion methods between species [16]. This dose is equivalent to 80 mg/day in humans, which falls within the therapeutic iron dose range of 80–200 mg/day [17].

### 2.5. Blood Sampling

At the end of experiments, rats were anesthetized with an intraperitoneal injection of a xylazine and ketamine mixture (10 mg/kg and 50 mg/kg, respectively) [18]. The abdominal cavity was exposed, and blood was withdrawn from the vena cava. Approximately 100 μL of blood was collected in a small Eppendorf tube containing 10 μL of EDTA solution for later hemoglobin assessment. 50 μL of blood was withdrawn from each animal in heparinized capillary tubes for the measurement of hematocrit values. Rats were euthanized by exsanguination through the abdominal aorta.

### 2.6. Biochemical Assessment

Drabkin's reagent was used to measure hemoglobin concentration spectrophotometrically by commercial diagnostic kits (Spectrum, Egypt). For serum preparation, clotted blood samples were centrifuged (500 g × 20 min). TIBC and serum iron levels were determined colorimetrically using commercial reagents (Cat. Number M11509i-23 for iron and Cat. Number M11554i-12 for TIBC. Biosystems, Spain). The sandwich enzyme immunoassay method was used to determine the serum ferritin (Rat Ferritin ELISA Kit. Cat. Number ER0947. FineTest, China) and hepcidin (Rat hepcidin ELISA Kit. Cat. Number ER1036. FineTest, China) levels.

### 2.7. Sample Size and Statistical Analysis

G power software was used to calculate the sample size (80% power, *α* = 0.05, effect size = 0.7), with serum ferritin level is chosen as the primary outcome. Despite 6 rats per group being acceptable, we included 8 rats per group to compensate for potential dropout 25%. Data are represented by the mean ± standard deviation (SD). Data were tested for normality using the K-S test. Comparisons of different outcomes were done using ANOVA test, followed by Tukey post hoc test, with significance level of *p* < 0.05 and a 95% confidence interval (SPSS software, version 22).

## 3. Results

The individual values for the measured parameters were included in a master table (See Supporting Information (available here)).

### 3.1. Effect of Oral Iron Treatment on the Blood Hemoglobin and Hematocrit Values

**Figure 1:**
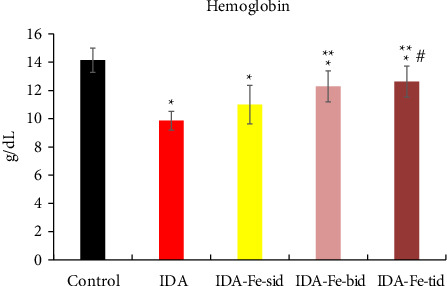
Hemoglobin levels among the studied groups. Each column represents the mean ± SD. IDA; anemic nontreated group, IDA-Fe-sid; single daily dose group, IDA-Fe-bid; twice daily dose group and IDA-Fe-tid; three times daily dose group. Statistical significance at *p* < 0.05, compared to the control (^∗^), IDA (^∗∗^), and IDA-Fe-sid (^#^).

**Figure 2:**
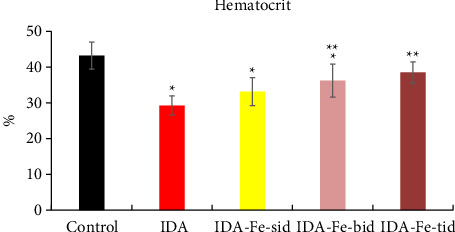
Hematocrit percentage among the studied groups. Data are represented as mean ± SD. IDA; anemic nontreated group, IDA-Fe-sid; single daily dose group, IDA-Fe-bid; twice daily dose group and IDA-Fe-tid; three times daily dose group, with statistical significance at *p* < 0.05, compared to the control (^∗^), IDA (^∗∗^), and IDA-Fe-sid (^#^).

The hemoglobin levels and the hematocrit percentage in the iron-deficient anemic nontreated rats (IDA) are significantly lower than the control nonanemic rats (*p* < 0.01), as shown in Table 2 and Figures 1 and 2. However, the administration of oral iron supplements resulted in different responses according to the frequency regimens. The anemic rats treated with oral iron preparation twice daily (IDA-Fe-bid) or three times daily (IDA-Fe-tid) showed significantly higher hemoglobin levels and higher hematocrit values, compared to the anemic nontreated animals (*p* < 0.05). On the other hand, the anemic rats treated with oral iron preparation once daily (IDA-Fe-sid) showed insignificant differences in both parameters compared to the anemic nontreated animals (*p*=0.326 for hemoglobin and 0.419 for hematocrit). No statistical significances are observed between the IDA-Fe-bid and IDA-Fe-tid groups (*p*=1.0).

### 3.2. Effect of Oral Iron Treatment on the Serum Iron and Ferritin Levels

**Figure 3:**
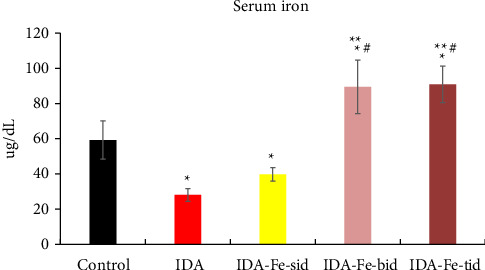
Serum iron levels among the studied groups. Anemic nontreated (IDA), and anemic treated rats with single dose (IDA-Fe-sid), twice daily doses (IDA-Fe-bid), and triple daily doses groups (IDA-Fe-tid). Data are represented as mean ± SD, with statistical significance at *p* < 0.05, compared to the control (^∗^), IDA (^∗∗^), and IDA-Fe-sid (^#^).

**Figure 4:**
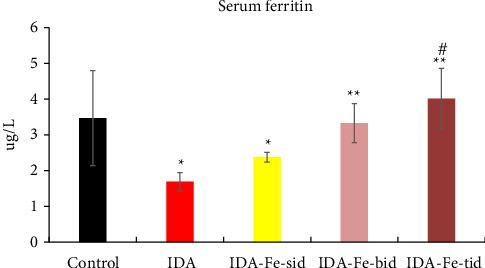
Serum ferritin levels among the studied groups. Data are represented as mean ± SD. IDA; anemic nontreated group, IDA-Fe-sid; single daily dose group, IDA-Fe-bid; twice daily dose group and IDA-Fe-tid; three times daily dose group. *p* < 0.05 is considered significance compared to the control (^∗^), IDA (^∗∗^), and IDA-Fe-sid (^#^).

As shown in Figures 3 and 4, the serum iron and ferritin levels in the nontreated anemic rats (IDA) are significantly decreased by nearly 50% compared to the control rats (*p* < 0.01). The single iron dose regimen did not produce significant change in both serum iron and ferritin levels over the 7-day experimental period (*p*=0.146, *p*=0.382, respectively), while split iron-dose regimens produced significant increments in both parameters compared to the IDA group (*p* < 0.01).

### 3.3. Effect of Oral Iron Treatment on the TIBC

**Figure 5:**
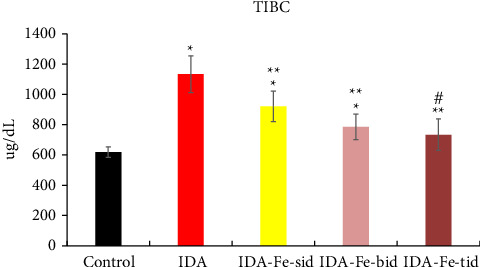
TIBC values among the studied groups. Data are represented as mean ± SD. IDA; anemic nontreated group, IDA-Fe-sid; single daily dose group, IDA-Fe-bid; twice daily dose group and IDA-Fe-tid; three times daily dose group. A statistical significance is considered at *p* < 0.05, compared to the control (^∗^), IDA (^∗∗^), and IDA-Fe-sid (^#^).

As shown in Figure 5, the TIBC values in the IDA group are significantly decreased compared to those of the nonanemic group (*p* < 0.01). The anemic rats treated with single or multiple doses of iron supplementation showed considerably lower TIBC values in comparison to the IDA group (*p* < 0.01). Furthermore, comparing the TIBC values of animals treated twice or thrice daily with oral iron revealed statistical insignificance (*p*=0.807).

### 3.4. Effect of Oral Iron Treatment on the Serum Hepcidin Level

**Figure 6:**
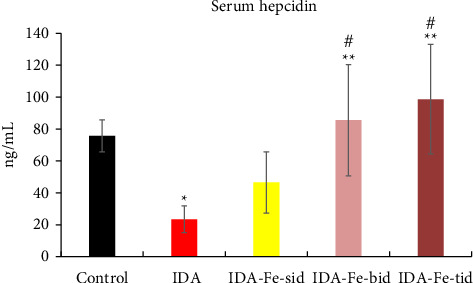
Serum hepcidin levels among the studied groups. IDA; anemic nontreated group, IDA-Fe-sid; single daily dose group, IDA-Fe-bid; twice daily dose group and IDA-Fe-tid; three times daily dose group. Data are represented as mean ± SD, with statistical significance at *p* < 0.05, compared to the control (^∗^), IDA (^∗∗^), and IDA-Fe-sid (^#^).

**Figure 7:**
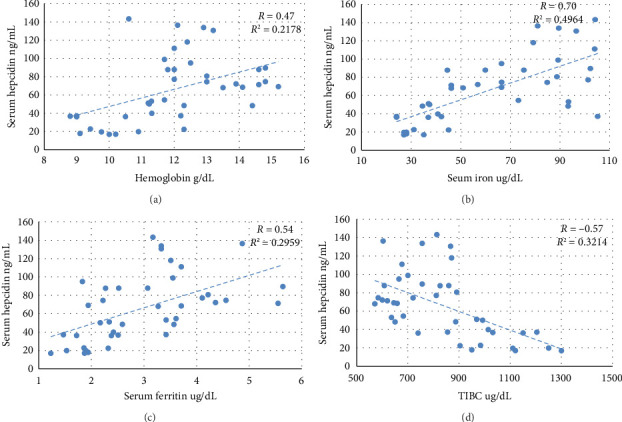
Pearson correlation chart of serum hepcidin. Serum hepcidin correlated positively with (a) hemoglobin, (b) serum iron, and (c) serum ferritin, and correlated negatively with (d) total iron binding capacity (TIBC).

The nontreated anemic animals (IDA) have serum hepcidin levels that are about one-third of those of the nonanemic animals (*p* < 0.01, Figure 6). The anemic rats treated with oral iron preparation twice daily and three times daily showed considerably higher serum hepcidin levels, compared to the IDA group (*p* < 0.01). In contrast, administration of oral iron once daily for 1 week did not show a significant difference in the hepcidin levels compared to the IDA group (*p*=0.330). Moreover, moderate positive correlations between serum hepcidin and hemoglobin concentration (*r* = 0.47), serum iron (*r* = 0.70), and serum ferritin (*r* = 0.54) are noticed. Conversely, hepcidin demonstrates a negative correlation with TIBC (*r* = −0.57, Figure 7).

## 4. Discussion

The present study aimed to evaluate three distinct iron replacement schedules for the treatment of IDA. It was shown that the twice and three times daily split doses could achieve a satisfactory, significant improvement in the iron plasma profile. Splitting the oral iron dose for 1 week successfully corrected the moderate IDA induced experimentally in rats, as evidenced by the significant increments in hemoglobin, serum Fe, TIBC, and serum ferritin levels. In addition, the hepcidin levels significantly increased to approach the control nonanemia group. A mild improvement of anemic parameters can still be seen in the animals treated with single iron intake; however, without statistical significance.

Due to well-known similarities between rats and humans, rats are commonly employed in preclinical research to evaluate both physiological and pharmacological responses. In this study, we use rats to establish a rapid and reproducible model of IDA. It is worth noting that iron administration for 7 days in rats is equivalent to 210 days of treatment in humans [19].

The technique used to induce IDA by repeated phlebotomy over 4 weeks succeeded in reaching a mean hemoglobin level of 9.95 g/dL, which is nearly the upper limit of moderate anemia. Another common method of inducing IDA in rats is feeding animals with an iron-deficient diet [20, 21]. The decision to adopt the bleeding technique has been made for two reasons: in the first place, the depleting iron from the diet may affect iron supplement absorption kinetics. Furthermore, the bleeding technique simulates the most common scenario of IDA, where iron is lost together with other blood components like transferrin, hepcidin, and proteins, all of which could affect iron homeostatic responses. Based on our previous pilot study of an animal model of experimentally induced IDA, we noticed that animals spontaneously recovered from anemia after 2 weeks when phlebotomy had been stopped and while animals were being fed the standard diet containing iron. This influenced the sampling timing of the study to be after 1 week only when animals are still suffering from anemia and the efficiency of iron supplementation can be assessed.

Ferrous sulfate is the prevailing drug among iron generics for the treatment of patients with IDA. Zero-order processes have been used to describe the absorption after extravascular iron preparation administration. Under zero-order absorption kinetics, a constant amount of iron can be absorbed per unit time, and it cannot be exceeded by increasing the dose [22].

Zero-order absorption kinetics could be explicated by the saturation of the transport systems after the administration of drugs, particularly at higher doses [23]. The zero-order absorption phenomenon may explain the failure of the once-daily schedule to improve anemia parameters.

Thus, while there might be a tendency to increase the ferrous sulfate dose to make sure that the maximum absorption rate is reached, such a strategy would usually result in a surplus of the iron compound to be present in the alimentary tract. Furthermore, iron preparations frequently produce nausea, vomiting, epigastric discomfort, constipation, and diarrhea [24]. These adverse effects result in a nonadherence to therapy in about half of anemic patients, thus averting effective management of their IDA. The unabsorbed iron can compromise the integrity of the intestinal epithelium by interacting with hydrogen peroxide, forming reactive hydroxyl radicals that may further have potential adverse effects on the gut microbiota [24].

Apart from the side effects caused by the excess iron in the gut, giving the preparation in a single daily total dose allows for the chance of absorption only during its gut transit time. Combined with its characteristic zero-order absorption kinetics, this makes a single dose inefficient in correcting the iron deficiency. In the current study, the once-daily dose did not succeed in significantly raising the anemia correction parameters. Dividing the dose over two or three administrations throughout the day, however, resulted in significant improvement.

To the best of our knowledge, very few studies have investigated the efficacy of splitting the oral iron dose. It is worth noting the report made by Stoffel et al. [25], in which they noticed that frequent iron administration in iron-depleted women did not affect iron absorption, despite the increments in serum hepcidin levels. An earlier study made by Moretti et al. [26] reported that iron absorption was equivalent between three doses (two in the mornings and one in the afternoon) and two morning doses in nonanemic young women, just over 24 h of iron intake. A recent meta-analysis concluded that both daily and alternative oral iron supplementation improve anemia parameters without superiority of one method to the other. However, the improvement was at a slower rate in the alternative regimen [26].

Siebenthal et al. conducted a randomized controlled trial involving 150 young women with IDA and reported similar findings regarding daily and alternative iron supplementation [27].

In contrast, a study conducted by Bulbul et al. on postmenopausal women and elderly men with IDA reported that hemoglobin increments were significantly greater with alternate-day dosing compared to split daily iron doses [28].

Of note, limited studies have directly compared different iron supplementation regimens in pregnant women. Stanworth et al. conducted a follow-up study on nonanemic pregnant women who received either daily or alternate-day iron supplementation from early pregnancy and concluded that daily dosing was associated with better adherence and may be more effective in preventing IDA during pregnancy than alternate-day iron dosing [29]. Similar findings were reported in an earlier study conducted by Adaji and his colleagues (2019), who demonstrated that both once-daily and twice-daily iron supplementation regimens were effective in preventing anemia throughout pregnancy [30]. In contrast, a recent randomized control by Lam et al. involving pregnant women with IDA found no significant differences in adherence or iron parameters between the daily and alternate-day iron supplementation groups [31].

In the current work, all iron supplementation regimens increased the circulating hepcidin level. Hepcidin levels exhibited a positive correlation with hemoglobin, serum ferritin, and serum iron, with the strongest association observed with serum iron. These findings suggest that serum iron level is the critical factor that determines hepcidin level.

Hepcidin negatively regulates the activity of the known cellular iron exporter, ferroportin [32]. Ferroportin mediates iron trafficking into the blood from the basolateral duodenal intestinal border, which absorbs dietary iron, from macrophages of the reticuloendothelial system that recycle iron from old erythrocytes, and from liver cells that store iron [32, 33]. Thus, hepcidin's main action is to lower serum iron levels by decreasing its intestinal absorption and by decreasing its release from iron stores.

On the other hand, erythroferrone, a key regulator of hepcidin production, has been overlooked in these studies. Erythroferrone is produced by erythroid precursors like erythroblasts in the bone marrow and acts directly on the liver cells to suppress hepcidin production, thus increasing the amount of iron available for red blood cell synthesis. Erythroferrone is responsible for hepcidin suppression during periods of increased erythropoietic activity governed by the erythropoietin hormone and allows for compensatory iron absorption during the recovery from blood loss-induced anemia [34, 35]. This suppression would play a key role in overcoming any possible temporary effect of iron supplementation on increasing hepcidin production, which retards iron intestinal absorption.

Improving intestinal drug absorption is one of the main reasons for developing the gastro-retentive drug delivery system (GRDDS). Trying to overcome the problem of poor oral bioavailability with some drugs, GRDDS tends to retain the drug in the stomach for a longer time, thus allowing slow release of the drug from the stomach to the duodenum [36]. Metformin is a good example of a drug having low bioavailability, absorbed in the duodenum and early part of the jejunum; therefore, several pharmaceutical companies developed GRRDS to improve its absorption [37]. Increasing the frequency of oral administration simply imitates GRDDS by increasing the time of drug delivery to the upper small intestine (duodenum and upper jejunum). The results obtained in this study support developing a novel GRDDS for iron.

A single daily administration of a drug seems to be more convenient for many patients compared to split dosing. A large-scale meta-analysis involving 8246 patients reported higher compliance with once-daily antibiotic regimens compared to multiple-dose regimens [38]. However, this may not be suitable in some situations, particularly when a single dose is associated with intolerability, side effects, or malabsorption, which can negatively impact adherence, prolong the treatment period, and may lead to treatment failure. In such cases, splitting the total dose into multiple smaller doses may mitigate these challenges. Our findings show that twice-daily iron dosing is as effective as three-times daily dosing in improving iron profile in IDA. In clinical practice, the twice regimen may be a more convenient approach for those who cannot tolerate the single daily full-dose regimen.

The improvement in anemic parameters achieved by the splitting doses was notably rapid and occurred within a mere week in the current work. This finding suggested that a split dosing regimen may combine the advantages of both the rapidity of the daily regimen and the efficacy of the alternative regimen. However, the adherence to this regimen in humans remains uncertain.

## 5. Conclusion

In the current work, splitting the daily oral iron doses successfully improved the IDA parameters and may be more efficacious than a single-dose schedule in iron deficiency anemic rats.

These findings contrast with those of some clinical studies involving both anemic and nonanemic patients, which reported that there were no significant differences between the two regimens. This discrepancy suggests that the optimal iron supplementation regimen remain a subject of ongoing debate, likely due to the multifactorial regulation of iron absorption.

Further clinical research on iron-deficient anemic patients is warranted to assess the efficacy of multiple iron doses in relation to patient adherence, the treatment duration, and the severity of anemia, taking into account the complex mechanisms governing iron absorption.

### 5.1. Study Limitations

Due to a shortage of the resources after the Corona pandemic, including immunohistochemical and PCR kits, we could not assess the hepatic ferritin and hepcidin. Other limitations include the lack of radioisotope iron preparation to allow more accurate tracing of the absorbed iron fraction. Furthermore, in the group that received the three-times dosing regimen, the timing spacing between the three doses was 6 hours instead of eight due to laboratory regulations regarding night working hours. Unfortunately, in the three-times dosing group, we were unable to determine whether 6 hour versus an 8-h interval influences iron absorption patterns, as this would necessitate tracing fractional iron absorption.

## Figures and Tables

**Table 1:** Animal groups, number, types, and treatment doses.

Groups	Number	Type	Iron dose mg/kg
Control	8	Rats	None
IDA	8	Rats	None
IDA-Fe-sid	8	Rats	7.1 (single dose)
IDA-Fe-bid	8	Rats	0.35 (twice/day)
IDA-Fe-tid	8	Rats	0.23 (thrice/day)

**Table 2:** Summary table for iron parameters.

Groups	Hb (g/dL)	Hematocrit (%)	Serum iron (ug/dL)	Serum ferritin (ug/L)	TIBC (ug/dL)	Serum hepcidin (ng/mL)
Control	14.15 ± 0.86 (CI:13.4–14.9)	43.25 ± 3.77 (CI:40.1–46.4)	59.23 ± 10.9 (CI:50.1–68.3)	3.47 ± 1.33 (CI:2.4–4.6)	618.88 ± 34.49 (CI:590–647.7)	75.73 + 10.08 (CI:67.3–84.2)
IDA	9.86 ± 0.67^∗^ (CI:9.3–10.4)	29.25 ± 2.71^∗^ (CI:27–31.5)	28 ± 3.63^∗^ (CI:25–31)	1.69 ± 0.25^∗^ (CI:1.5–1.9)	1133.50 ± 121.48^∗^ (CI:1031.9–1235.1)	23.35 + 8.40^∗^ (CI:16.3–30.4)
IDA-Fe-sid	10.99 ± 1.37^∗^ (CI:9.9–12.1)	33.13 ± 3.91^∗^ (CI:29.9–36.4)	39.70 ± 3.9^∗^ (CI: 36.4–43)	2.38 ± 0.133^∗^ (CI:2.3–2.5)	920.50 ± 101.1^∗^(^∗∗^) (CI:836–1005)	46.50 ± 19.16 (CI:30.5–62.5)
IDA-Fe-bid	12.29 ± 1.09^∗^(^∗∗^) (CI:11.4–13.2)	36.25 ± 4.62^∗^(^∗∗^) (CI:32.4–40.1)	89.50 ± 15.2^∗^(^∗∗^)^#^ (CI:76.8–102.2)	3.33 ± 0.55(^∗∗^) (CI:2.9–3.8)	785.00 ± 83.71^∗^(^∗∗^) (CI:715–855)	85.57 + 34.83(^∗∗^)^#^ (CI:56.5–114.7)
IDA-Fe-tid	12.63 ± 1.11^∗^ (^∗∗^)^#^ (CI:11.7–13.5)	38.50 ± 3.02(^∗∗^) (CI:36–41)	90.94 ± 10.37^∗^(^∗∗^)^#^ (CI:82.3–99.6)	4.02 ± 0.84(^∗∗^)^#^ (CI:3.3–4.7)	733.50 ± 104.21(^∗∗^)^#^ (CI:646.4–820.6)	98.70 + 34.33(^∗∗^)^#^ (CI:70–127.4)

*Note:* IDA; anemic nontreated group, IDA-Fe-sid; single daily dose group, IDA-Fe-bid; twice daily dose group and IDA-Fe-tid; three times daily dose group. Data are represented as mean ± SD, (95% CI: lower-upper), with statistical significance at *p* < 0.05, compared to the control (^∗^), IDA (^∗∗^), and IDA-Fe-sid (^#^).

## Data Availability

The data that support the findings of this study are available from the corresponding author upon reasonable request.

## Supporting Information

Supporting Information 1 Supporting figure graphic abstract: Schematic representation of the study design illustrating the four anemic groups subjected to biweekly phlebotomy over 4 weeks to induce iron deficiency anemia: nontreated iron-deficient group (IDA) and three treatment groups receiving daily oral iron once (IDA-Fe-sid), twice (IDA-Fe-bid), or three times (IDA-Fe-tid). At the end of the study, blood samples were collected for the assessment of hemoglobin (Hb), hematocrit, serum iron, total iron-binding capacity (TIBC), serum ferritin, and hepcidin levels.

Supporting Information 2 Supporting table: Raw data of the anemia parameters: hemoglobin (Hb), hematocrit, serum iron, total iron-binding capacity (TIBC), serum ferritin, and hepcidin levels.
