# Supplementary figures and images for: Split Daily Oral Iron Dosing Enhances Correction of Iron-Deficiency Anemia in Rats
Source: Anemia. 2025 Jun 28;2025:9976840. doi: 10.1155/anem/9976840 (PMC12257470; doi:10.1155/anem/9976840)

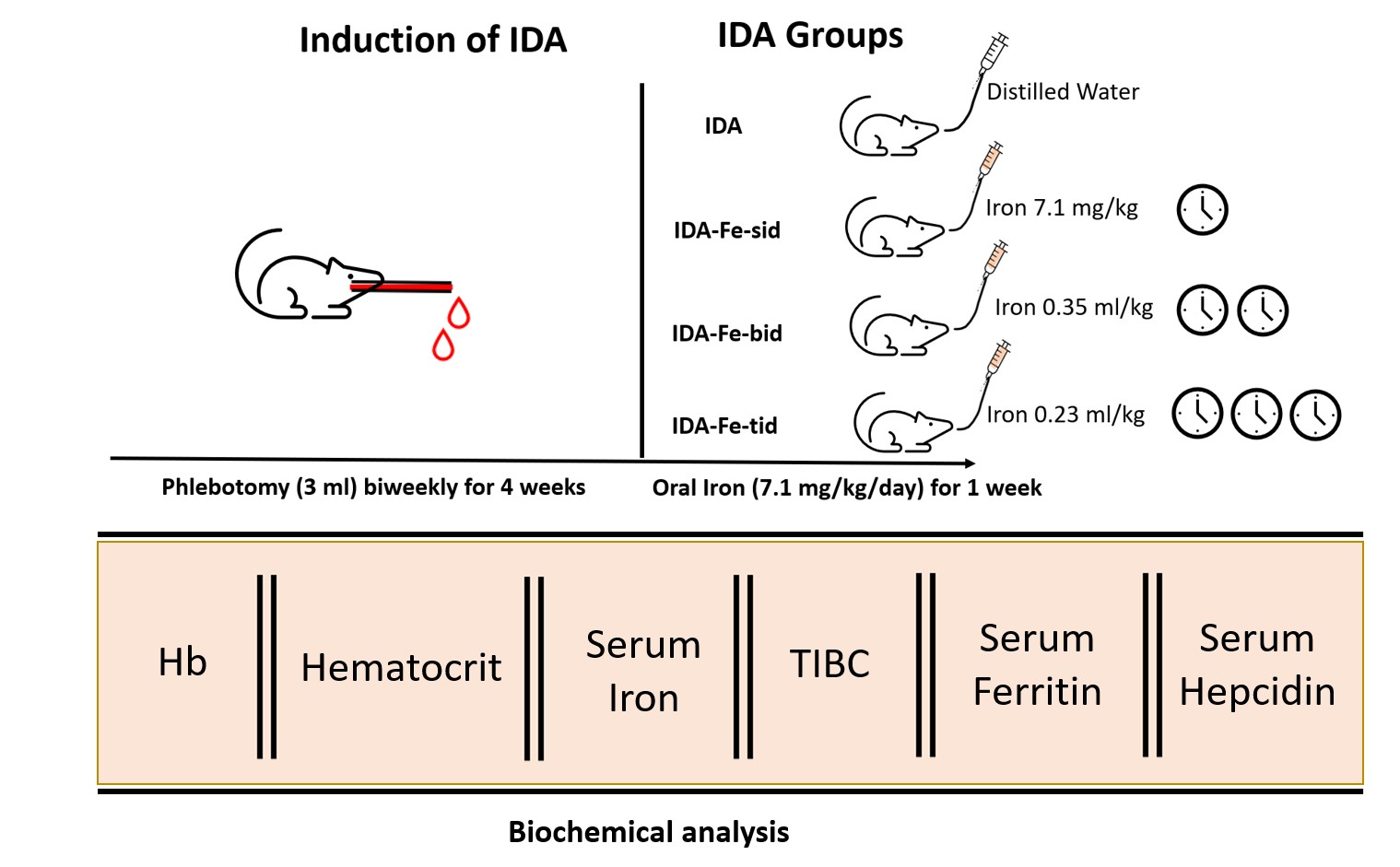

Supplement: Supporting Information 1 — Supporting figure graphic abstract: Schematic representation of the study design illustrating the four anemic groups subjected to biweekly phlebotomy over 4 weeks to induce iron deficiency anemia: nontreated iron-deficient group (IDA) and three treatment groups receiving daily oral iron once (IDA-Fe-sid), twice (IDA-Fe-bid), or three times (IDA-Fe-tid). At the end of the study, blood samples were collected for the assessment of hemoglobin (Hb), hematocrit, serum iron, total iron-binding capacity (TIBC), serum ferritin, and hepcidin levels. [file 9976840.f1.jpg]
