# Supplementary material for: Split Daily Oral Iron Dosing Enhances Correction of Iron-Deficiency Anemia in Rats
Source: Anemia. 2025 Jun 28;2025:9976840. doi: 10.1155/anem/9976840 (PMC12257470; doi:10.1155/anem/9976840)
Supplement: Supporting Information 2 — Supporting table: Raw data of the anemia parameters: hemoglobin (Hb), hematocrit, serum iron, total iron-binding capacity (TIBC), serum ferritin, and hepcidin levels. [file 9976840.f2.pdf]

	Hb g/dL	hematocrit %	iron ug/dL	TIBC ug/dL	ferritin ug/L	Hepcidin n[illegible]
Control						
1	12.50	39.00	66.29	665	1.83	94.96
2	14.60	46.00	46.06	620	5.55	71.19
3	15.20	49.00	66.29	645	1.94	68.97
4	14.60	40.00	75.28	608	2.52	87.79
5	13.50	40.00	46.06	571	3.27	67.89
6	13.90	41.00	56.71	600	4.36	72.1
7	14.10	47.00	50.82	658	3.71	68.37
8	14.80	44.00	66.33	584	4.56	74.55
IDA						
1	10.20	30.00	35	1120	1.87	16.93
2	9.10	25.00	28	950	1.94	17.85
3	10.00	32.00	27	1300	1.23	16.81
4	9.00	27.00	24	1204	1.47	37.06
5	10.50	31.00	24	1150	1.72	36.18
6	10.90	33.00	28	1250	1.53	19.81
7	9.42	28.00	31	984	1.86	22.62
8	9.76	28.00	27	1110	1.9	19.57
IDA-Fe-sid						
1	12.30	33.00	34.47	887	2.59	48.38
2	12.30	36.00	44.94	904	2.32	22.26
3	11.20	36.00	36.89	969	2.34	51.05
4	8.80	27.00	42.06	1032	2.51	36.71
5	11.80	35.00	44.49	826	2.27	87.69
6	9.00	27.00	36.81	740	2.38	36.11
7	11.23	35.00	37.29	992	2.17	50.03
8	11.31	36.00	40.65	1014	2.42	39.8
IDA-Fe-bid						
1	12.40	37.00	79.1	871	3.51	117.91
2	10.60	30.00	104.01	813	3.17	143.23
3	13.00	41.00	84.67	720	2.22	74.32
4	12.00	39.00	59.76	860	3.07	87.79
5	12.20	34.00	105.01	855	3.42	37.1
6	14.40	43.00	93.11	650	3.57	48.27
7	11.70	31.00	89.1	700	3.55	98.83
8	12.00	35.00	101.2	811	4.11	77.1
IDA-Fe-tid						
1	12.10	37.00	80.85	603	4.87	136.28
2	14.80	44.00	102.09	756	5.64	89.52
3	12.90	36.00	89.33	756	3.33	133.8
4	11.70	38.00	73.11	682	3.61	54.59
5	11.30	40.00	93.29	636	3.42	53.12
6	13.20	40.00	96.38	867	3.33	130.63
7	12.00	34.00	103.81	677	3.71	111.04
8	13.00	39.00	88.62	891	4.22	80.61
